# An *in-situ* participatory approach for assistive robots: methodology and implementation in a healthcare setting

**DOI:** 10.3389/frobt.2025.1648737

**Published:** 2025-10-16

**Authors:** Ferran Gebellí, Raquel Ros

**Affiliations:** 1 PAL Robotics, Barcelona, Spain; 2 Artificial Intelligence Research Institute (IIIA-CSIC), Bellaterra, Spain

**Keywords:** human-robot interaction, participatory design, i*n-situ* Co-design, assistive robots, longitudinal study, in-the-wild study, healthcare application

## Abstract

**Introduction:**

This paper presents a participatory design approach for developing assistive robots, addressing the critical gap between designing robotic applications and real-world user needs. Traditional design methodologies often fail to capture authentic requirements due to users’ limited familiarity with robotic technologies and the disconnection between design activities and actual deployment contexts.

**Methods:**

We propose a methodology centred on iterative *in-situ* co-design, where stakeholders collaborate with researchers using functional low-fidelity prototypes within the actual environment of use. Our approach comprises three phases: observation and inspiration, *in-situ* co-design through prototyping, which is the core of the methodology, and longitudinal evaluation. We implemented this methodology over 10 months at an intermediate healthcare centre. The process involved healthcare staff in defining functionality, designing interactions, and refining system behaviour through hands-on experience with teleoperated prototypes.

**Results:**

The resulting autonomous patrolling robot operated continuously across a two-month deployment. The evaluation through questionnaires on usability, usage and understanding of the robotic system, along with open-ended questions revealed diverse user adoption patterns, with five distinct personas emerging: enthusiastic high-adopter, disillusioned high-adopter, unconvinced mid-adopter, satisfied mid-adopter and non-adopter, which are discussed in detail.

**Discussion:**

During the final evaluation deployment, user feedback still identified both new needs and practical improvements, as co-design iterations have the potential to continue indefinitely. Moreover, despite some performance issues, the robot’s presence seemed to generate a placebo effect on both staff and patients, while it appears that staff’s behaviours were also influenced by the regular observation of the researchers. The obtained results prove valuable insights into long-term human-robot interaction dynamics, highlighting the importance of context-based requirements gathering.

## 1 Introduction

Assistive robots can potentially perform many of the complex tasks required for beneficial applications (Matarić and Scassellati, 2016). In health and social care, they can both support medical staff and patients in hospitals and care homes (Ohneberg et al., 2023). The implementation of novel service robots typically involves a process of design, development and piloting. However, further continuity to real adoption is generally minimal, not to mention non-existent. When discussing with healthcare providers, serving both as technology suppliers and adopters, a primary reason for this limitation is that the functionalities of the robots do not align effectively with the practical daily routines of the users (Yoo et al., 2024). Throughout the design process, development decisions might have been wrongly taken because the reality of how the users will end up using the system has been overlooked. Users’ requirements are typically gathered at a very early stage in the product development process. At this initial stage, most users are not even familiar with robots, and thus they cannot provide the necessary feedback to truly capture the system requirements (Rasmussen et al., 2024).

HRI researchers have previously identified similar concerns and proposed alternatives to traditional design processes by introducing in-context co-design at various stages. An early contribution by Šabanović et al. (2014) advocates for *in-situ* evaluation of early prototypes. Although their work primarily focuses on the evaluation phase, they iterate the design process to reflect on insights gained through the *in-situ* evaluation. User involvement with robot prototypes at earlier stages in the process is further explored in two notable works. The immersive participatory design approach by Olatunji et al. (2024) embeds a robot within a user’s home, enabling customisation alongside the family. The method involves direct co-designing with users immersed in their environment, shortening the design-prototype-evaluate-redesign iteration loop. In contrast to our approach, their focus lies in prototyping customised specific robot tools and interfaces, while conceptualisation activities are conducted through semi-structured interviews rather than low-fidelity prototyping, as in our work. It is worth mentioning that participants in their study had extensive experience with robots, eliminating the need for prior familiarisation with robot capabilities prior to concept generation, a requirement common in other use cases. In this context, Stegner et al. (2023) proposes a situated participatory design method comprising three phases. First, exploring the technology’s capabilities, selecting and designing scenarios, and enacting them through the use of real technology. Next, these scenarios are repeatedly wizarded (i.e., the robot is remotely controlled by researchers) in realistic settings to validate their relevance. And finally, the designs are discussed with stakeholders. This interactive approach effectively identifies functionality and interaction design through direct user engagement. However, since the method does not include system deployment, it lacks iteration over the realistic technical implementation of the system.

Our proposed approach integrates elements from the aforementioned methods into a comprehensive *in-situ* co-design process. Conceptualisation and user requirements emerge from an initial phase involving the use of a simplified version of the potential solution. This allows users to engage not only with tangible concepts but also to experience them in context, enriching their feedback with specific and actionable insights. These insights directly inform functionality and interaction design, shaping system behaviour toward deployment and evaluation.

In this work, we present the implementation of our proposed approach at “El Carme”, an intermediate care service part of Badalona Serveis Assistencials (BSA), a publicly owned healthcare organisation in Spain. More specifically, we worked in collaboration with the orthogeriatric ward, which admits primarily elderly, highly vulnerable patients with multiple pathologies and orthopaedic conditions, who need to be treated for several weeks or months, typically after undergoing varied interventions. The stakeholders’ starting premise was that the assistive robot shall support the healthcare staff within the context of risk assessment of patients during their stays at the hospitals. With this goal, we planned a series of co-design sessions with the healthcare staff, involving *in-situ* prototyping, requirements gathering and refinement and finally, and a 2-month in-the-wild experimentation stage to pilot the outcome of the participatory design process.

The main contributions of this work are:

• A participatory design approach for conceptualisation, development and deployment of robots, centred on iterative *in-situ* co-design to maximise the likelihood of context-based requirements definition and refinement.
• An implementation of the proposed approach at an intermediate healthcare centre over a 10-month period.
• A pilot evaluation of the resulting solution, reporting on the system’s performance and user perception after 2 months of 24/7 usage.

The paper is organised as follows. Section 2 overviews related works in the area of participatory design of robotic systems in hospital settings through longitudinal studies. Section 3 describes the participatory design approach proposed along with its implementation, while Section 4 introduces the outcome of the robotic system developed throughout the process. Section 5 describes the materials and methods used for the evaluation pilot carried out at the hospital. The outcomes of the deployment after 2 months of usage are presented in Section 6. Finally, Section 7 provides a discussion on the results and limitations of the current work, while Section 8 concludes it providing future research lines.

## 2 Related work

### 2.1 Longitudinal and in-the-wild studies in HRI

Research has highlighted the need for longitudinal studies in human-robot interaction, as users typically have limited prior experience interacting with robots. Understanding how people interact and perceive robots, both during (Smedegaard, 2019) and after (Belpaeme, 2020) the novelty effect, merits further investigation. Despite calls to address the novelty effect and conduct longitudinal research, the review from Baxter et al. (2016) encountered that only 5 out of 96 HRI studies included more than one interaction per user. Longitudinal studies are challenging to implement due to difficulties in identifying suitable use cases, recruiting participants, and securing the necessary resources (Leite et al., 2013), while wizard-of-oz techniques are impractical (Kunze et al., 2018). Additionally, users increasingly expect some degree of personalisation (Kunze et al., 2018; Leite et al., 2013), which introduces further ethical and privacy concerns according to Jokinen and Wilcock (2021).

Longitudinal studies often require unsupervised interactions with autonomous robots, with various levels of ecological validity: online studies, laboratory studies, and field studies conducted “in the wild” (Hoffman and Zhao, 2020). While online studies offer scale and efficiency, participants may lack engagement (Zhou and Fishbach, 2016), and the absence of physical interaction with robots undermines the validity of results (Bretan et al., 2015). Alternatively, research is typically conducted in laboratory environments where participants interact with real robots. While such settings afford control over variables and support the establishment of causal links, the longitudinal factor is quite limited (to only a few interactions), interactions are forced, and the samples often consist of university students, limiting the generalisability of findings to broader, real-world contexts (Hoffman and Zhao, 2020). Therefore, as Jung and Hinds (2018) points out, our understanding of human responses to robots in real-world settings remains limited. The dominance of lab-based (and online) research is largely due to the practical challenges associated with field studies, ranging from technological limitations to resource constraints Belpaeme (2020).

Although infrequent, few studies in HRI are performed both in a longitudinal and in-the-wild manner. De Graaf et al. (2016) deployed 70 commercially available social robots in participants’ homes for up to 6 months. The study revealed a mere-exposure effect, whereby repeated interaction with a novel stimulus leads to more positive evaluations as familiarity increases. Another study (Jeong et al., 2023) introduced a robotic coach that delivered positive psychology interventions to college students. A week-long on-campus deployment indicated improvements in students’ wellbeing, mood, and motivation, though the short duration limited longer-term insights. Hofstede et al. (2025) conducted a multi-country field study with 90 older adults, caregivers, and formal carers during two-to six-week home deployments. The study identified six key considerations—personalisation, interactivity, embodiment, ethical issues, connectedness, and dignity—with personalisation and interactivity emerging as most critical. Finally, Zafrani et al. (2024) presented a six-week study with 19 older adults that examined the use of a socially assistive robot for physical training, highlighting that assimilation is heterogeneous and shaped by individual experiences.

### 2.2 Assistive robots in hospital settings

For the past 2 decades, assistive robots have been entering hospitals to provide support to caregivers, patients and/or family members and visitors. Their tasks have ranged from more routine jobs, such as delivering items–medication, meals, samples, etc.,– or monitoring patients’ vital signals, to welcoming, entertaining and facilitating communication with users (Ohneberg et al., 2023).

However, few of the assistive systems targeting hospital settings have been actually tested in the field and for long periods, or have been used in practice. Within the scope of robots for healthcare settings, Kirschling et al. (2009) ran a 125-day pilot of a robot delivering medication in two patient care units at a tertiary care academic medical centre. An early work to provide medical care for patients with infectious diseases using teleoperated robots was performed by Kraft and Smart (2016), but within a simulated Ebola Treatment Unit. After the COVID-19 outbreak, assistive robots were regarded as an opportunity to support healthcare staff and isolated patients. First developments and testing in lab-based scenarios emerged to supply goods, medications, and to facilitate communication between the staff and infected patients (Thamrongaphichartkul et al., 2020; Yoo et al., 2024). A 2-and-a-half-month deployment in a hospital was carried out by Bartosiak et al. (2022). The semi-autonomous robot was used to mediate communication between staff and patients in confined areas, while monitoring patients’ vital signals through observation of clinical devices. Other solutions include the usage of autonomous mobile robots for the disinfection of hospital facilities (Zaman et al. (2022).

Besides academic studies, two commercial robots have been successfully deployed in hospitals to actively support healthcare workers. Moxi, developed by Diligent Robotics^1^, is a hospital assistant robot that helps clinical staff by performing logistical tasks such as delivering supplies, fetching equipment, and transporting lab samples. Several care facilities in the U.S. have reported the success of the system for prolonged periods of time (e.g., between December 2021 and 2 April023^2^) and performing over 150.000 trips for pharmacy staff^3^. Despite such accomplishments, no reasons have been reported for discontinuing the use of the robot. On the other hand, Lio, created by F&P Personal Robotics^4^, is a multifunctional service robot for care assistant tasks (Mišeikis et al., 2020). Equipped with a robotic arm and voice interaction capabilities, Lio can assist with daily activities like opening doors, reminding patients to take medication, and even engaging in simple conversations to reduce loneliness. Case studies have been carried out in Germany and Switzerland, some operating for over a year. Tasks are discussed with the stakeholders and functionalities are adapted accordingly, but we could not find reports on how this process is actually done.

The development of assistive robotics has often been driven by the critical needs of healthcare systems in our society. Challenges such as chronic staff shortages, the burden of repetitive and time-consuming tasks, the demand for greater efficiency in patient care, or even disease outbreaks, have pushed the need for this technology. However, as pointed out in an early work by Mutlu and Forlizzi (2008), who performed an intermittent longitudinal 15-months ethnographic study of an existing autonomous delivery robot, it is essential to consider how these innovations influence the workflow of clinical staff, reshape their roles, alter their working environment, and affect their social dynamics–both among colleagues and in their interactions with patients and informal caregivers. To ensure that such technologies truly support and enhance healthcare delivery, the use of value-driven, participatory design methods is essential. These approaches prioritise the real-world needs, values, and experiences of end-users, enabling the development of systems that are not only usable and effective but also meaningful and empowering for those who rely on them daily (Mutlu and Forlizzi, 2008; Bartosiak et al., 2022). In the next subsection, we delve into the literature of such participatory design methods.

### 2.3 Participatory design in social robotics

Participatory design, often used interchangeably with co-design (Sanders and Stappers, 2008), involves the active involvement of diverse stakeholders–such as end-users and domain experts (Axelsson et al., 2021) – throughout the design process. Individuals without robotics expertise can meaningfully participate in shaping the robots’ design (Lee et al., 2017), playing a pivotal role (Schuler and Namioka 1993) in the iterative construction of the system. Participatory design methods support a mutual-shaping dynamic between robotics technologies and the societies in which they are embedded (Šabanović (2010). This means that society influences the way robots will behave, but also the introduction of robots modifies the society itself.

In practice, participatory design has been applied across various HRI contexts. For instance, it has supported the co-design of social robots aimed at enhancing adolescent mental health (Björling and Rose, 2019), helping cognitively impaired citizens (Rodil et al., 2018) or assisting older adults experiencing depression (Lee et al., 2017). These studies leverage an array of participatory techniques, including card-sorting (Ostrowski et al., 2021), role-playing (Björling and Rose, 2019), and prototyping (Björling and Rose, 2019; Ostrowski et al., 2021). However, as reviewed in Rogers et al. (2022), the most common techniques used are workshops, followed by focus groups and interviews, while concept generation through drawing, storyboard or card sorting is less used. The application of these methods typically lacks actual contact with physical robots. Therefore, considering that users have little or no experience with robots, it is often difficult for them to provide valuable feedback about the potential use of these technologies in their everyday lives. With this aim, several works have proposed alternative methods that emphasise the need for more active involvement in co-designing with tangible technologies across different aspects and stages of the process. Liberman-Pincu et al. (2024) introduce CoDeT, a toolkit centred on the aesthetic elements of assistive robots. Stegner et al. (2023) propose Situaded Participatory Design (sPD) with a focus on concept and interaction design of systems through wizarded prototypes. Their methodology is implemented in a senior living facility to explore how a robot could assist residents in manipulation tasks. Olatunji et al. (2024) present an immersive approach that highlights co-design with the robot-specific features. In their use case, users had an extensive experience with assistive robots, allowing conceptualisation through interviews. Their study involved a two-week design phase focused on custom tools for an assistive robot, followed by a two-week deployment phase. An end-to-end PD approach is presented in Winkle et al. (2021), in which the behaviour of a coaching robot and an educational robot is learnt through an *in situ* online teaching phase with a domain expert.

In this work, we present a comprehensive participatory design methodology, which integrates an evolving robot prototype throughout all stages of the process, including conceptualisation and functionality definition, interaction design, and system behaviour. The method was implemented and evaluated over a 10-month period in an intermediate healthcare centre. A first overview of the envisioned approach was presented in Gebellí et al. (2024a), where the overall methodology was initially outlined. A detailed description of the *Interaction Design* step (described in Section 3.2) with a focus on explainability was subsequently introduced in Gebellí et al. (2024b). Finally, a thorough analysis of the users’ understanding of the system over time is provided in Gebelli et al. (2025). This work presents an in-depth account of the entire participatory design approach, encompassing its conceptual framework, practical implementation, and the results obtained. Unlike previous publications that have focused on an isolated component or specific results on partial evaluations, this paper offers an integrated perspective that captures the full scope and evolution of the methodology.

## 3 A participatory design approach

One clear limitation in designing robotic technologies is the constrained (if any) shared contextual knowledge among the different participants involved in the design process. This includes differences in expertise, surrounding environments, working conditions, and more. On the one hand, end-users and key stakeholders are typically unfamiliar with robotic technologies and the features or capabilities they may offer. On the other hand, research and development (R&D) teams are often unaware of the users’ everyday routines in which the system is intended to be used.

Existing methodologies address the latter gap by conducting co-design sessions where user needs are gathered through ethnographic studies, interviews, focus groups, and similar methods, as reviewed in Section 2. While these are valuable sources of information, we argue that they often suffer from a key limitation^5^: they are typically conducted outside the actual context of use (with the exception of ethnographic studies). Frequently, co-design activities take place in environments disconnected from the deployment setting–such as meeting rooms–or outside the users’ routines, which prevents the enactment of realistic scenarios that provide valuable feedback during the design process (Šabanović et al., 2014; Olatunji et al., 2024; Stegner et al., 2023; Winkle et al., 2021).

To overcome these limitations, we propose a participatory design methodology that combines co-design techniques with *in-situ* requirements acquisition through prototyping in pre-pilots. In this approach, all participants (stakeholders and the R&D team) are co-located within the actual context of use, working with an early, simplified prototype of the potential solution to jointly explore its possible applications. This setup enables all participants to develop a well-informed understanding of the artefacts, environmental dynamics, and use cases from the outset, and to maintain this understanding throughout the refinement phases, ultimately maximising the effective development of robotic systems designed to support daily routines.

**FIGURE 1:**
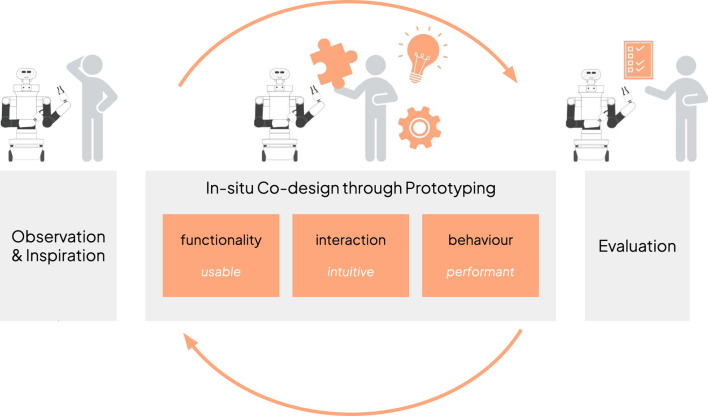
Overview of the participatory design process for robot design, underscoring the proposed *in-situ* co-design approach and its relation with the expected key attributes of the system: usable, intuitve and performant.

Figure 1 illustrates the proposed approach divided into three main phases:

• *Observation and inspiration*: The goal of this phase is to define the scope of the project, initiate engagement with stakeholders and understand the problem. Techniques such as interviews and observations are essential to gather insights that will drive the next phase. In this work, this phase was rather short, since we were departing from a previous experience with the healthcare centre in a sister project. More specifically, we carried out an initial meeting with the Head of Innovation of the hospital to get to know each other and to define the scope of the project. Next, three researchers visited the healthcare centre to get first-hand knowledge of the environment, dynamics and routines of an ordinary day. Finally, a larger meeting with the Head of Innovation, the Head of Nurses and the Main Geriatrician of the unit took place to do an initial brainstorming of possible functionalities, plan the next steps of the co-design project, and set the logistics.
• *In-situ Co-design through Prototyping*: This phase corresponds to the core of the proposed approach, where co-designing with end-users merges ideation, co-creation and prototyping activities within the real context of use. Based on the insights obtained in the previous phase, a low-fidelity prototype is prepared to work hand-by-hand with users in defining the functionalities–what it should do–, interactive capabilities–how it should interface with users–and behaviour of the system–how to achieve the tasks. By clearly identifying and prototyping these features, we argue that we can achieve usable, intuitive and performant robotic systems to effectively support people in real settings.
• *Evaluation*: The final phase corresponds to the evaluation of the outcome of the previous phase through longitudinal in-the-wild pilots.

The steps in the proposed method can be repeated as many times as necessary until a testable version of the evolved prototype is obtained. While constant feedback is gathered within the second phase for different parts of the system–supporting frequent iterations–, a complete assessment of the system is not achieved until the end.

We next describe the steps involved in the *In-situ Co-design through Prototyping* phase, which constitute two of the main contributions of this work. For each step, we introduce its methodology alongside its implementation, using a real use case to demonstrate its practical application. The *in-situ* co-design process carried out at the intermediate healthcare centre “El Carme” in Spain lasted 8 months. The final phase, *Evaluation*, lasted 2 months and it is introduced in Section 5.

### 3.1 Functionality definition

The main goal of this step is to identify the functionality of the robot system, i.e., the task(s) to perform based on the context of use. One of the main shortcomings we often face when working in robotics projects is that users are quite unfamiliar with robots, and hence, there is a high mismatch between their expectations and the real capabilities of robots. Based on the idea of mutual-shaping Šabanović (2010), we claim that providing a minimally functional tool enriches the co-design process, since users can start shaping their ideas into actionable artefacts based on understanding of current technologies’ advantages and limitations and make informed decisions. At the same time, researchers can immediately witness how users expect to be supported by the technology in their everyday by, first understanding the need for a given feature, and then, re-creating simple prototypes and testing *in situ* with the users as ideas develop by actual use.

To this end, a low-fidelity prototype of the system is prepared in this step. In the context of robotics, we argue that a low-fidelity prototype should correspond to a teleoperable robot [similar to Stegner et al. (2023)], where basic capabilities are prepared based on the initial insights gathered in the *Observation&Inspiration* phase. In this work, that first phase led to the identification of the following potential tasks the robot could perform:

• Monitoring: The robot patrols all the rooms, or a subset of them (e.g., rooms in which patients should not leave the bed or chair because of their high risk of falling). The robot should detect falls (a person on the floor) within visible areas and alert the staff. Alerts are received by all staff, and it is up to them to decide the action to take. It is critical to report all the potential falls, prioritising a very high recall over precision, as far as the false positive alarms are not excessively intrusive and uncomplicated to dismiss, as informed by the staff. Moreover, the system should work during nighttime, when patients are less assisted and the risk of unnoticed falls is higher.
• On-demand tasks: The staff should be able to send the robot to a specific location when requested, so they can “observe” a given situation through the robot’s cameras. All the staff members can request such checks, but the head nurse should be able to set priorities.
• Logistics: (1) The robot should bring items within the unit among the staff (e.g., blankets, medicines) and (2) the robot should bring items across units, i.e., from the pharmacy, which is accessible by a lift; and between units, when a unit is out of stock of given items. The robot should be provided with a tray or bag to transport the items. It might need to be locked with a code depending on the nature of the item. For some medicines, special permission would be required.
• Miscellaneous functionalities: video calls with the external world; support rehabilitation exercises; identify people who should not be at specific places at given times (e.g., visitors outside visiting hours or accessing restricted areas).

A first low-fidelity prototype was developed during the following 3 weeks. Considering the above tasks, three essential developments were carried out:

1. The robot platform: we opted for a PAL Robotics TIAGo on a differential base, which was already available in the research team. The robot provides basic functionalities, such as motor control, navigation, speaking, playing sounds and controlling the base LEDs. A small transparent box was attached to the back of the robot tray to emulate a carrying basket for delivering items. A tablet was also added to communicate the internal state of the robot and for users to introduce the required information to fulfil a given task when needed.
2. Teleoperation interface: A simple application was developed for researchers to manually trigger various robot behaviours. These included the playback of audio messages, sounds and LEDs, the initiation of movement patterns, and the logging of relevant information at any point. This application enabled the emulation of different requested functionalities, i.e., patrolling, remote check, delivery, and charging the battery. This interface allowed for rapid prototyping and flexible adaptation of application flows in response to participant feedback and situational demands. A tablet device was used to host the interface, offering portability and ease of use during *in-situ* co-design. The robot’s movements were teleoperated with a separate gamepad controller.
3. Mobile application: A simple mobile app was available for staff members to receive notifications of events detected and to request on-demand tasks, such as remote checking of a room or delivery of items. The phones were connected to the teleoperation interface in the tablet, so researchers would remotely receive task requests from the staff and trigger changes in the mobile phones’ displays.

**FIGURE 2:**
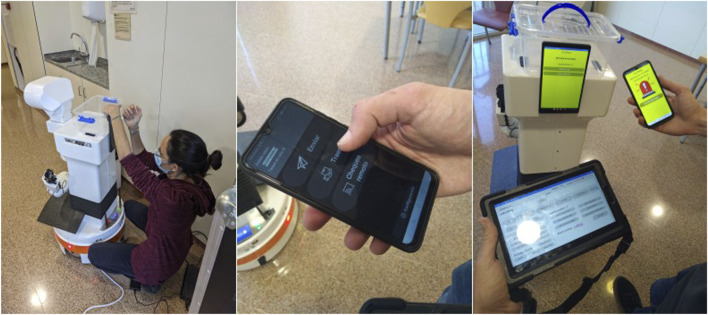
Low-fidelity prototypes of the tools developed for the functionality definition: (left) a researcher attaching a tablet on the robot to facilitate communication with users; (middle) a mobile phone app for nurses to make requests; (right) the TIAGo robot, mobile phone app and teleoperation interface to run the co-design of system functionality.

Figure 2 illustrates the different tools developed for and during the *functionality definition* step.

Once the prototype was ready, we spent 1 week at the care centre teleoperating the system to understand the real use of the system and discuss the functionalities with the main stakeholders. By doing so, we can either confirm or reject initial ideas derived from the initial brainstorming. Moreover, those that are confirmed can be further refined considering the exact use in different circumstances. This is one of the main critical inputs that we are seeking at this stage, since often researchers do not realise such details unless they are performing the task. Requests could either come from the staff themselves or from the research team, who could spontaneously identify a situation where the robot could provide some sort of support.

We spent 4 h per day at different times of the day during 1 week. We covered the workflow from 9 a.m. to midnight and both on weekdays and weekends. Staff workload and patient care can greatly vary from 1 day to another, especially on weekends. Moreover, the staff also varies, and thus, reaching different voices was essential. Notes and comments were either voice-recorded or written in a document for further reference.

**FIGURE 3:**
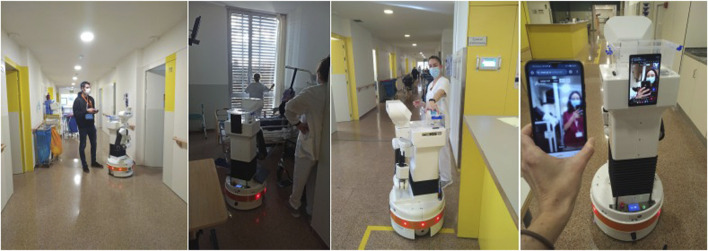
*In-situ* teleoperation with the prototype. From left to right: a researcher teleoperating the robot in the patrolling task; the robot monitoring a patient’s room; a nurse delivering an item in the robot’s basket; setting a video call to test communication between staff and patients.

During this activity, the *in-situ* teleoperation with the prototype, the staff was very busy, so we opted for applying the “shadowing” technique, i.e., following the staff with the robot, although staff could still make requests to the prototyped system on their own. By doing so, we could jointly point out any situation where the robot could provide support and test it through teleoperation. At the same time, this was an opportunity to stay next to the staff, learn about their everyday routines, open discussions on how the robot could better assist, or come up with additional ideas. On their side, it was also useful for them to understand the current limitations of the technology, thus setting the right expectations about what a robot could or could not do eventually. Two additional tasks were incorporated at this stage: deliveries to patients and calls between staff and patients. Figure 3 exemplifies different teleoperated tasks during the *in-situ* teleoperation.

Patients in the unit were not explicitly included as part of the process. However, they were directly or indirectly involved anyway, especially after introducing the two additional tasks. In any case, their response was generally open, curious and looking forward to having a robot around working for real.

Based on the experiences gained from the *in-situ* teleoperation, an analysis of the tasks performed was conducted to determine which ones should proceed to the next step of the co-design. Several constraints were imposed: the task should highly support the staff routines; the system should run fully autonomously; the system should be easy to use and seamlessly integrated in the working environment; the system should be implemented in 5 months.

Given the above constraints, the research team and the main stakeholders agreed that the best option was to focus on a single task to make sure it ticked all the requirements. As such, the chosen task was the patrolling routine, given the great impact it would have in terms of assisting the staff to enhance patient safety by proactively identifying hazardous situations, such as fall detection. It was agreed that the robot would continuously monitor the rooms configured by the staff at given times, and it would trigger alarms after detecting a fallen patient on the floor, which the staff would receive and manage in a phone app. The robot would also trigger alarms for not-in-bed patients, to mitigate potential falls, and closed-door rooms, to ensure staff maintain constant visibility of patients inside their rooms. These alerts would only trigger for a group of vulnerable patients identified by the staff. With the functionality of the robot system completed, the next step is to co-design the interaction between users and the system.

### 3.2 Interaction design

This step focuses on how stakeholders, primarily the end-users, interact with the system. A workshop is first organised to identify critical interaction situations observed during the previous step and that require further refinement. Based on the outcomes of the workshop, the interfaces are prototyped and refined *in-situ* with the users.

**FIGURE 4:**
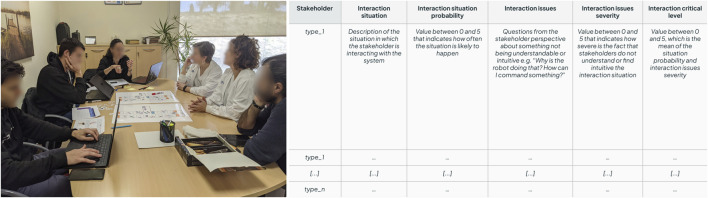
Interaction design workshop where role-playing activity took place with the main stakeholders (left); interaction table template to identify pain points when interacting with the system (right).

Through a role-playing session (Figure 4 left), we reviewed a first draft of proposed interaction flows to identify those situations where the system’s interface and behaviour might be unclear. To collect such pain points, we employed what we call the *interaction table* (Figure 4 right), which structures the co-design of intuitive interactions by defining:

• Stakeholder, indicating the stakeholder type.
• Interaction situation, describing the interaction instance, i.e., what the users and robotic system are specifically doing.
• Interaction situation probability, a value between 0 and 5 representing the probability of an interaction taking place. Higher values indicate more frequent situations.
• Interaction issues, including aspects that stakeholders may not understand or may not find intuitive. We recommend formulating questions from the stakeholder’s viewpoint to bring these ideas into a tangible form.
• Interaction issues severity, a value between 0 and 5 representing the severity of a problem in an interaction. Higher values indicate a higher degree of non-understanding or non-intuitiveness.
• Critical level, combines the probability and severity fields. We recommend computing it as the mean of probability and severity, but for certain applications, different functions might be more adequate, such as the maximum value.

The *interaction table* can be further extended to cover additional features, such as explainability strategies to incorporate into the system, as presented in Gebellí et al. (2024b).

Similarly to the previous step, we next *in-situ* iterate the interfaces with the users through low-fidelity prototypes and mock the interactions to gather feedback on the different measures identified in the *interaction table*. Several iterations should be conducted to refine the interaction table and update the interaction issues and critical level, according to the received feedback. Moreover, additional unforeseen situations should be included when necessary. The number of interactions might be large, and it might be unfeasible to have time to test them all. The critical level allows for prioritising the *in-situ* tested interactions, focusing on the ones with the highest priority. Furthermore, we recommend starting with the stakeholders who mostly interact with the system, which are the most frequent users.

In this work, we organised an *in-situ* testing to refine the configuration of the robot’s patrolling routine and alarm handling interfaces throughout a day, from 10.00 to 18.00, to cover two staff shifts. The most critical interaction tasks to refine were: to configure the patrolling routines of the robot, to update them based on different events (e.g., a new patient arrives at the ward) and to manage the alarms triggered by the robot. Since the interfaces to perform these tasks were done through the mobile phone, we did not bring the robot to the ward and only used two mobile phones, which were handed to the users at different times of the day. To mock up a large set of situations, we used our laptops to recreate patrolling configurations (6 different situations) and triggering alarms (8 different situations). We repeated the procedure 4 times throughout the day, each round with different users. After each round, we collected their feedback, and when possible, we would quickly modify the interfaces based on their suggestions to test the different options.

### 3.3 Behaviour design

After the functionality and interaction design have been agreed, the next step is to design the behaviour of the robot and to implement it. A first version of a high-fidelity system is carried out in the lab, where the focus is centred around fulfilling the functional and interaction requirements gathered in the previous steps. Nevertheless, in these type of development projects, it is often the case that researchers also need to collect real data either to train or to validate the models developed. Hence, we went back to the hospital on two different occasions. First, to evaluate different sensors that could potentially feed the perception sub-system to identify falls and the presence of staff within the rooms. More specifically, we took an RGBD camera, a thermal camera, RFID (Radio-Frequency Identification) tags and BLE (Bluetooth Low Energy) beacons. And second, we brought the robot to build the map of the environment and to refine the navigation strategies to overcome all sorts of obstacles present in such a dynamic environment. With this additional data, informed decisions were made regarding the technologies to use in the system and to refine its implementation with realistic data.

**FIGURE 5:**
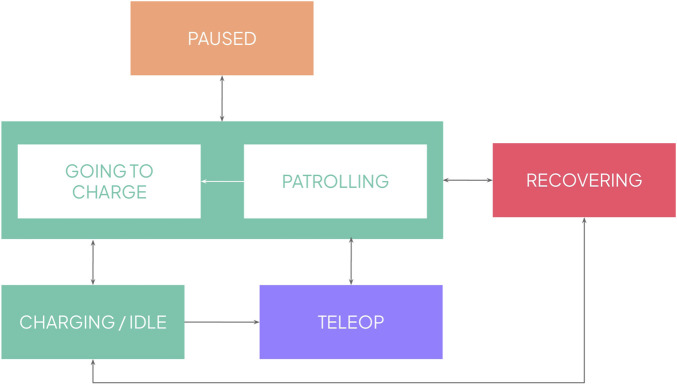
Overall state machine of the behaviour of the robot.

The overall behaviour of the robot is depicted in Figure 5. We first focused on the patrolling routines themselves, where the robot is waiting in the charging unit until a new patrolling routine starts. At that point, the robot leaves its charging unit and navigates through the ward, visiting the configured rooms that require monitoring. Once the patrolling routine is finished, it goes back to its charging unit. After iterating with the staff, we identified additional states that the robot should include: *pause*, necessary for those situations where the staff needs the robot to briefly pause its routine, but resume it afterward maintaining its current schedule; and *teleop*, to manually move the robot, specially necessary when the robot gets completely lost and thus, needs to be guided to its charging unit to relocalise itself. The additional *recovery* state was also added during the *in-situ* refinement, which corresponds to situations where the robot detects an internal failure and needs to recover autonomously (e.g., not receiving images at the right frame rate, which only requires restarting the camera). During this time, the system shall stop its routine and resume it as soon as it has recovered. These situations were only present when the robot was patrolling for longer periods of time, which were not experienced in the lab tests.

Once a complete system is ready, a last iteration takes place where the full system is thoroughly tested in the context of use. This last iteration allows for *in-situ* fine-tuning of any aspect of the system, either from an interaction or system performance perspective, maximising the success of the deployment in the evaluation phase. In this work, we spent 4 days before the evaluation period started to refine the system, mainly devoted to tuning the perception thresholds to trigger the alarms, adjusting autonomous navigation speeds and safety margins, and ensuring communication between the different devices throughout the ward.

## 4 System overview

**FIGURE 6:**
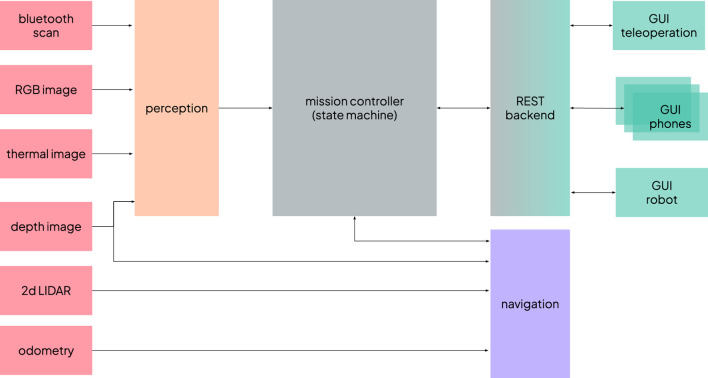
Overview of the system architecture and links between the main modules.

In this section, we provide a technical outline of the system implementation. Figure 6 provides an overview of the main modules. We have used ROS (Robot Operating System) Noetic for most of the modules, except for the graphical interfaces, which communicate with the rest of the system via a REST interface.

### 4.1 Perception

The perception module is responsible for supplying the information required by two of the alarms that the system can trigger: the fallen person alert and the not-in-bed patient alert. It operates in parallel on input from two camera streams–one thermal and one RGB. The thermal camera is particularly important for ensuring reliable operation during nighttime conditions, a requirement gathered during the co-design; moreover, it also performs effectively during daylight hours, especially in backlighting situations. The RGB camera complements the thermal stream, enhancing the robustness of person and posture detection during the day.

**FIGURE 7:**
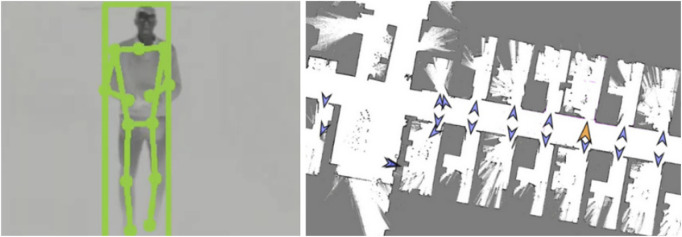
Thermal camera image with the body keypoints (left); top-down map of part of the facility with the waypoints in front of the doors (right).

The perception pipeline comprises several stages. First, human body keypoints are detected in either the thermal and RGB 2D images using Google MediaPipe pose landmark detection^6^, as visualised in Figure 7, left. Next, depth information is integrated to reconstruct the 3D positions of the skeletal landmarks, focusing primarily on the hips and shoulders. These 3D keypoints are then referenced against the estimated floor plane to determine their height. Based on this information, a heuristic-based classifier labels each detected person as either fallen, standing or unrisky posture.

A detected fallen posture would always trigger an alarm, with no further filtering. In contrast, a not-in-bed patient alarm required further checks: either the person is alone in the room (i.e., no other human figures are detected by the pipeline) or there is no nearby nurse present. To assess the proximity of nursing staff, the system estimates the location of their mobile devices by leveraging Bluetooth signal strength. A model was calibrated to convert RSSI (Received Signal Strength Indicator) into distance values to update a particle filter tracking the devices’ positions.

### 4.2 Navigation

For autonomous navigation within the hospital environment, we employed the standard ROS navigation stack. The robot relied on a 2D laser sensor for localisation, and it was augmented by the depth image for obstacle avoidance. This multi-sensor fusion was especially critical in cluttered environments, such as hospital corridors, where mobile medical equipment (e.g., carts and trolleys) often presented partial occlusions that the laser scanner alone could misinterpret or overlook. An initial map of the unit was constructed and validated (Figure 7 right) to ensure reliable localisation and navigation, confirming that the robot could maintain positional accuracy without the need for additional localisation aids such as fiducial markers.

Rooms included a set of preconfigured waypoints: a position in front of the door, an entry point just past the door, and one or more “scanning points.” At each scanning point, the robot would halt and perform a series of head movements to search for fallen or standing patients. Given the variability in room layouts, the number and placement of scanning points were tailored to each specific room. The robot would activate its perception system to detect falls and not-in-bed patients only in those scanning points, avoiding triggering alarms while navigating to reduce the number of false positives. Moreover, the navigation system provided the necessary information for the closed-door alarms. If the robot failed to compute a viable path from the position in front of the door to the initial entry waypoint inside the room, the door was considered to be closed.

To optimise safety and manoeuvrability, different navigation parameters were applied depending on the context: reduced maximum speed and narrower obstacle margins were used within rooms, while more relaxed parameters were set for corridor traversal.

### 4.3 Graphical user interfaces

**FIGURE 8:**
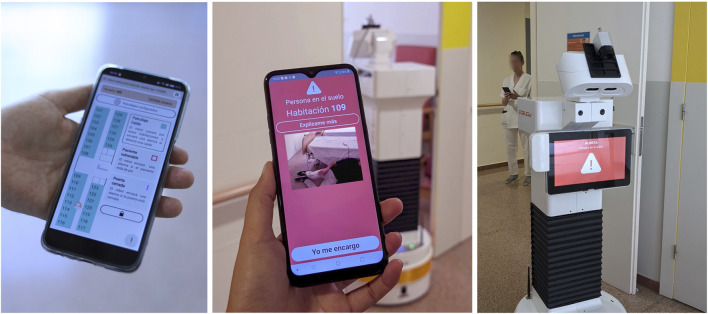
Mobile interface with a top-down view map where the different rooms can be configured, and the live position of the robot is seen (left); mobile phone, while triggering an alarm, with an image of the detected situation (middle); the chest display of the robot during an active alarm (right).

The mobile interface consisted of an Android application installed on multiple smartphones. It offered two core functionalities: (1) configuration of patrolling routines (Figure 8 left) and (2) reception and handling of alerts (Figure 8 middle). Each user had to log in at the start of the shift, which allowed us to process individual usage data. For patrol configuration, users could schedule multiple rounds, each defined by a start and end time and a selection of rooms to be patrolled. Patrols could be set to repeat daily or be executed once. While the fallen person alert was always active for the selected rooms, the not-in-bed and open door alarms could be selectively activated. When an alert was triggered, the mobile device responded with visual, auditory, and vibration signals, depending on the severity of the event. The alert screen provided detailed information about the incident and allowed users to press a button to indicate that they were addressing the issue, so it would stop in all other phones. Additionally, the application displayed real-time information about the robot’s current operational state, battery level, and location, and provided controls to pause or resume the patrol.

Moreover, the application allowed users to request explanations in order to obtain further information about why a recent alarm had been triggered or clarifications on how to proceed to fix an issue. This was done through a button available right after addressing an alert, which displayed two context-relevant question-answer pairs.

The onboard robot interface (Figure 8 right) was implemented as a simplified display located on the robot’s chest screen. It provided a continuous, high-level overview of the robot’s operational status, including whether it was patrolling, paused, charging, navigating to a charging station, or issuing an alert.

Privacy protection was a core requirement in the design and implementation: (1) images are temporarily transmitted only to authorised staff smartphones when required (i.e., only when an alarm is triggered) and never stored; (2) there is no voice interaction; (3) and the system does not process any personal or medical information. These safeguards ensured that patients were not directly identifiable.

## 5 Materials and methods

This section presents the participants, procedure and distributed questionnaires for the evaluation later reported in Section 6. The aim is to assess the adoption of the robotic system developed through the participatory design process described in the previous sections in the everyday routine of the staff.

### 5.1 Participants

Participants were recruited from the nursing staff of the orthogeriatric unit of the BSA healthcare centre^7^. A total of N=31 participants took part in the study, comprising 27 females and 4 males, including 9 nurses and 22 nursing assistants. The nursing staff operates in four shifts: morning, afternoon, and two alternating night shifts, with 7–8, 4-5, and 3-4 members working simultaneously during each respective shift. Throughout the study, we recruited a total of 12 morning-shift, 5 afternoon-shift, and 14 night-shift staff members. Participants had different presence levels throughout the study. Some staff members had planned holiday breaks, and were replaced by substitutes during their leaves. All participants provided written consent for their voluntary participation after receiving a detailed briefing about the study.

### 5.2 Procedure

After providing informed consent, participants completed a first questionnaire, the *understanding questionnaire*, prior to any interaction with the system. Subsequently, a hands-on tutorial session was conducted for each shift to introduce the system’s functionality and primary features. The robot was present during the session, and all participants were allowed to interact with the system. Immediately following the tutorial, participants completed once again the *understanding questionnaire* as well as the *usability questionnaire*. This marked the beginning of the robot’s deployment. The same procedure was repeated for each subsequent shift.

Following deployment, the system operated autonomously, 24 h a day, 7 days a week, for 5 weeks. Researchers intervened only to address an autonomous navigation issue during the first week and to collect completed questionnaires throughout the pilot. In addition to the *understanding questionnaire* and the *usability questionnaire*, the *usage questionnaire* was administered during selected weeks. Starting from the middle of week 3, there was a progressive introduction of patients from an adjacent unit within the healthcare centre, the palliative unit^8^. Consequently, both patients and some staff were relocated to the unit where the robot was deployed, which added more activity to the ward compared to the previous weeks. The transferred patients were generally in a more critical condition, and hence, more visitors were also present. An additional tutorial session took place for those staff members who joined during the transfer of palliative patients.

**FIGURE 9:**
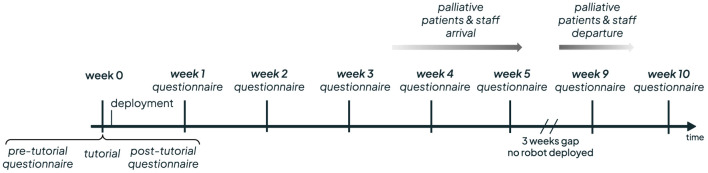
Timeline of the deployement weeks.

Furthermore, after week 5, there was a 3-week break due to a hardware issue that required repairs, compounded by a scheduled holiday break for the research team. Finally, the autonomous deployment continued for 2 more weeks. A mean of 13.5 participants answered the questionnaires at each weekly round^9^. The full timeline can be seen in Figure 9.

### 5.3 Measures

We automatically collected several performance and usage metrics. The daily patrolled hours and kilometres were recorded, as well as the configured and visited rooms. The timestamped triggered alarms were recorded jointly with their type, which participant addressed them and if a further explanation was requested. Within the mobile application, the amount of time that the app was running with the screen on was stored per user.

Apart from the automatically collected data, the following questionnaires were distributed to the participants. The *understanding questionnaire* evaluates the user’s understanding of the robot using both subjective and objective measures. Detailed evaluation on *understanding* and its dynamics in HRI is reported in Gebelli et al. (2025). Subjective understanding, i.e., user-perceived and self-rated level of system understanding, was assessed using a 7-point scale in response to the statement: “My level of understanding of the robot is…”. Objective understanding, i.e., the actual comprehension of the system (Rong et al., 2023), was measured using a set of six multiple-choice questions covering the robot’s behaviour, such as “What will the robot do after a “closed door” alarm?” or “How does the robot detect a standing person?”. Responses were scored as 0 or 1 depending on correctness. Both scores were normalised to range from 0 to 1. The questionnaire was distributed every week.

The *usability questionnaire* was implemented by the System Usability Scale (SUS) administered immediately following the tutorial, and during weeks 2, 3, 4, 5, 9, and 10. The SUS provides a score between 0 and 100 to evaluate perceived usability.

Similarly, we distributed the *usage questionnaire* to evaluate the frequency of use of the system. A 5-point Likert scale was required in response to the question: “How often did you use this technology?”. Participants also responded to two open-ended questions: (a) “What are the main reasons for the frequency of use?” and (b) “How could the technology be improved or what would have to happen for you to use it more often?”. The questionnaire was answered the weeks 1, 3, 4, 5 and 9, to gather data on initial use, full mid-use and use at the end of the pilot.

All questionnaires were administered individually in a think-aloud format with a researcher present to gather potential insights into the people’s reasoning and to resolve any doubts they could have.

## 6 Results

In this section, we present the results of the two-month autonomous deployment of the system at the healthcare intermediate centre.

### 6.1 System performance

Throughout the deployment, the robot operated autonomously for a total of 327.8 h and covered 78.2 km. This corresponds to a daily average of 6.7 h and 1.6 km. The operation was distributed across 641 patrolling rounds, during which the robot inspected a total of 7,656 rooms. These figures indicate a high level of use, suggesting that the system was sufficiently robust for long-term autonomous operation.

**FIGURE 10:**
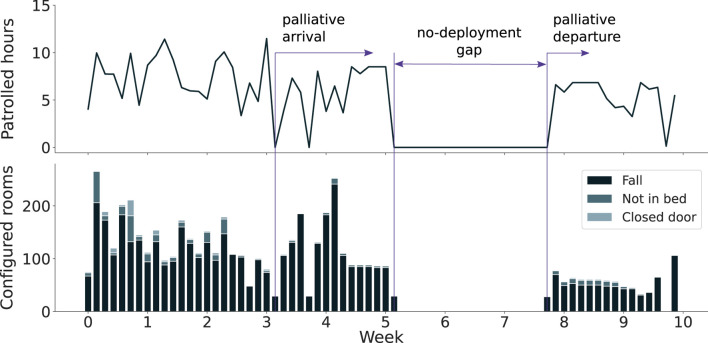
Daily autonomous patrolling hours (top) and accumulated configured rooms (bottom) throughout the deployment period.

As illustrated in Figure 10, while there is a slight decrease in usage following the initial weeks, the system maintained a relatively consistent level of activity over time. Between the third and the fourth weeks, the palliative patients and staff were introduced. This shift in patient profile, along with the arrival of unfamiliar staff, resulted in a noticeable drop in system usage. Despite the interruption in usage between weeks 5 and the end of week 7, usage levels returned to normal following the break.

When comparing the patrolled hours and the number of configured rooms, we observe that for a similar number of patrolled hours, the robot was configured to survey a smaller number of rooms during the last weeks of deployment. A possible reason could be that the nursing staff learned how to better use the system and configure only the rooms where it was truly needed, e.g., due to high risk of falls. Moreover, it can also be observed how the majority of the rooms were configured to trigger only fall alarms, and only a subset included the not-in-bed or door alarms. This measure corresponds to the low number of high-risk patients present in the ward.

Nevertheless, one would expect that with the palliative patients’ arrival, more rooms would include the not-in-bed or door alarms active. However, on the one hand, we corroborated with the participants that given that more staff and visitors were present in the room due to the patients’ fragile conditions (weeks 4 and 5), the need for having the robot patrol highly vulnerable patients was less required. On the other hand, the presence of new staff, who were less experienced and more sceptical of the role of the robot in palliative care, also diminished its use. A similar observation on substantially different perceptions and use of the robots by two different wards was identified in Mutlu and Forlizzi (2008), raising the questions on how differences in work practices, social context and use of the physical environments may impact adoption of such technologies.

Table 1 summarises the triggered alarms during the deployment. The first block refers to failure-related events. Specifically, navigation alarms denote instances in which the robot failed to recover autonomously and required staff intervention to remove obstacles. If the robot remained non-functional after removing the obstacles, staff were instructed to press the emergency button (equivalent to releasing the robot motors) and manually return it to the charging dock. The emergency button was also used for immediate emergency stops, though such cases were extremely rare. The activation of the emergency button was spread across the deployment, with a significantly higher amount during the first weeks, when the navigation failures were more common. In some instances, users performed a full system restart to reinitialise the robot when persistent autonomous behaviour issues occurred.

**TABLE 1:** Summary of the triggered alarms, true/false positives and requests for explanations. The first block (top) refers to failure-related alarms, while the second block (bottom) includes perception-related alarms.

Triggered alarms	Total	True positives	False positives	Explanation requests
navigation alarms	225	–	–	27
emergency button presses	45	–	–	7
full restarts	36	–	–	–
fall alarms	14	0	14	0
not-in-bed alarms	5	2	3	0
closed door alarms	68	28	40	1

The second block of Table 1 reports on the perception-related alarms. True and false positive rates were based on nurses’ input via the mobile application following an alarm. Alarm detection performance was low, with a high number of false positives. It should be noted, however, that closed-door alarm values are not fully reliable, as some early reports were inversely logged due to confusion among staff on certain shifts. Only three real fall incidents occurred, though the robot was either idle or patrolling other rooms, and therefore could not be detected by the system.

The final column of Table 1 presents the total amount of requested explanations by the participants, which were available through a button when the respective alarms had been triggered, as explained in Section 4. Upon computing the ratio of explanation requests in relation to the triggered alarms, categorized into failure-related alarms (comprising navigation errors and emergency button activations) and detection-related alarms (including falls, not-in-bed, and closed doors alarms), it becomes evident that explanations were solicited by users in ∼ 12% of the cases involving failure alarms, whereas for detection alarms, explanations were solicited in only ∼ 1% of the cases. This suggests that users actively request explanations primarily when corrective interventions regarding the robot’s behaviour are necessary from their side. Conversely, when explanations pertain to the robot’s detection performance, such as justifying false positive detections, users exhibit less inclination to request them. Actually, some participants verbalised these ideas when talking about perception-related alarms, e.g., P07 said that “If you explain it to me, that’s fine, but if not, I’m not interested either.”

### 6.2 Overall user perception

**FIGURE 11:**
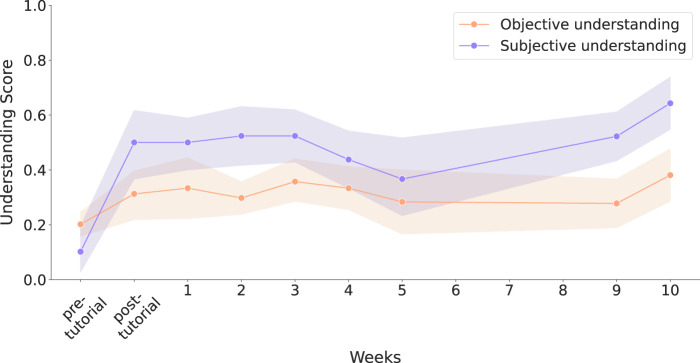
Evolution of subjective and objective understanding scores across the deployment. Vertical bands indicate 95% confidence intervals.

Regarding the understanding metrics, represented in Figure 11, a statistically significant improvement was found in both measures when comparing scores before the tutorial and at week 10 (subjective: p<0.001 ; objective: p=0.004 ). We employed the Mann–Whitney U test since the data did not follow a normal distribution (validated through a Shapiro-Wilk test) and samples were not paired. Before the tutorial, there was no significant difference between subjective and objective understanding scores (p=0.09) , suggesting that participants had a reasonably calibrated self-assessment. However, by week 10, a significant difference was present (p=0.01) , indicating a decoupling of the two measures. The Wilcoxon test was used for the comparison between objective and subjective understanding, as the data was not normal, but it was paired. These results suggest participants tended to overestimate their understanding of the robot as the deployment progressed. Notably, during weeks 4 and 5 —when palliative patients and new staff were introduced—both understanding measures declined, but a sharper decrease in subjective understanding caused the gap between them to narrow.

**FIGURE 12:**
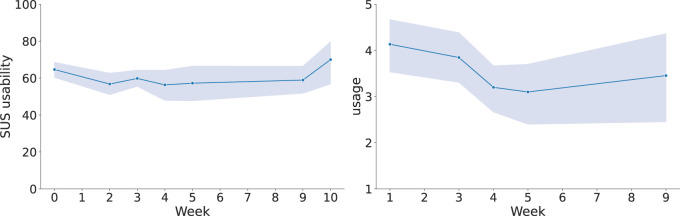
Evolution of the SUS average scores (left) and reported usage (right) across each week. Vertical bands indicate 95% confidence intervals.

Concerning the usability and usage metrics, the results in Figure 12 illustrate how both the SUS usability and usage scores followed a similar trajectory. Specifically, average scores decreased initially but later recovered, returning to levels similar to those observed at the beginning of the deployment. As the data was not normally distributed, confirmed by a Shapiro-Wilk test, and the sample was unpaired across weeks (due to varying participants), we used the Mann–Whitney U test for statistical comparisons. For SUS usability scores, there was a statistically significant decrease between week 0 (post-tutorial) and week 2 (p=0.037) , whereas the difference between week 0 and week 10 was not statistically significant (p=0.084) . Regarding reported technology usage, a significant drop was observed between week 1 and week 4 (p=0.023) . This decrease coincides with the arrival of new patients and staff from other units. However, the comparison between week 1 and week 9 was not significant (p=0.230) .

### 6.3 Individual user evaluation

**FIGURE 13:**
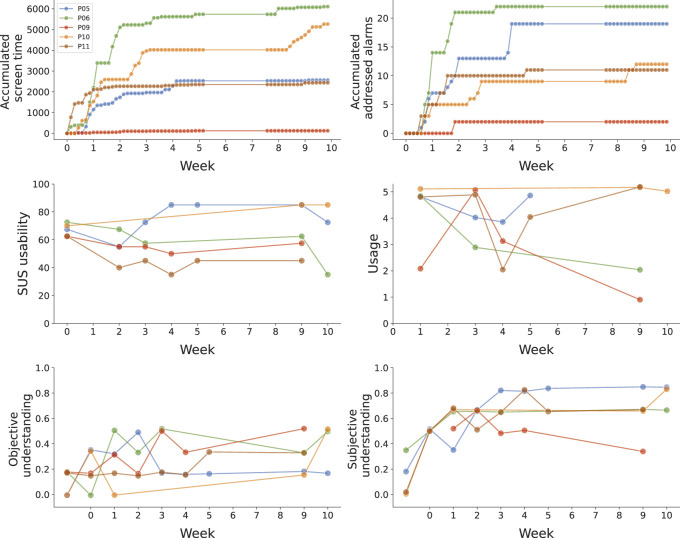
Individual user outcome trajectories over time across six dimensions: accumulated screen time, accumulated addressed alarms, SUS usability, self-reported usage, subjective understanding, and objective understanding. Vertical jitter has been applied to better observe the differences for overlapping scores.

In addition to aggregated analyses, we examined the behaviour of individual users to identify representative usage profiles and extract qualitative insights. Based on a combination of quantitative metrics and qualitative responses–from the questionnaires and think-aloud responses–, we identified five participants who constitute a prototype of user personas presented below. Figure 13 shows the individual evolution for each profile of real system use through the screen-on time for the app, which is a metric of general usage for both configuration and alarms addressing, and the number of addressed alarms, which helps to notice the users who were rather passive in the system usage. The figure also includes the evolution of the responses for the usability and usage questionnaires, reported subjectively by participants, and both the objective and subjective understanding metrics. These personas allow for individual tracking of the evolution over time for a single participant, as aggregated metrics might hide some interesting trends. The variability across participants in the metrics can be explained by several factors. On the one hand, differences in staff roles and routines across shifts influenced the interaction with the system. On the other hand, individual differences in acceptance and familiarity with technology also played a role. The available data is not sufficient for a quantitative analysis of the factors that contribute to these discrepancies, but the qualitative individual evaluations highlight the need for tailored strategies. Possible solutions include targeted training sessions for specific roles, incremental introduction of functionalities and adaptive interface features.

The following profiles were distinguished:

• “Enthusiastic high-adopter”: P10 consistently engaged with the system throughout the deployment. This user actively configured patrol routines and responded to alarms. P10 expressed excitement about the technology and demonstrated growing trust and comfort with the system over time. P10 reported high usability (SUS) and frequent usage, with both metrics increasing. Understanding scores—both objective and subjective—also improved during the study, indicating a genuine interest in learning about and leveraging the robot’s capabilities. Finally, the verbal responses also showed excitement with the platform: “We cannot be there all night, but the robot can” or “it works well, it’s an additional monitoring tool we have”. This persona represents an excited adopter who likes the system and actively uses it.
• “Disillusioned high-adopter”: P06 showed strong initial engagement by frequently configuring patrols and addressing alarms, as mentioned to the team “I’ve been playing with it, in case 1 day I’m on my own. We’ve used it every day since you left”. Early in the deployment, P06 reported high usability and usage, as well as high objective and subjective understanding, suggesting confidence in managing the system. However, over time, the perception of the system deteriorated as the user experienced system limitations or unmet expectations. This resulted in a strong decline in SUS and usage scores. This decrease in usage is, however, partially related to the palliative patients’ arrival, as P06 recognised “We’re with the other palliative care unit, and there’s a lot of fuss. Now that they’re leaving, we’ll be able to use it more”. This user illustrates a persona that is initially an “enthusiastic high-adopter” and transitions to the “unconvinced mid-adopter” explained below.
• “Unconvinced mid-adopter”: P11 used the system frequently, but appeared to do so out of an external push from the hospital management rather than genuine interest from the very beginning. The user consistently reported one of the lowest SUS scores from the beginning, reflecting scepticism about the system’s value. P11 also verbalised in the open questions that the system was not very performant: “It should stop less often and detect people better” or “we had to stop it due to failures”. Moreover, the participant did purposely lie on the floor to test the robot’s capabilities, but the failure in detecting the faked fall further decreased trust, as reported by the participant “we no longer have confidence in it because we tried to see if it detected us on the floor and it failed”. Interestingly, P11’s subjective understanding was high—suggesting confidence in the knowledge of the robot—yet this was not supported by objective scores, which remained low. Nevertheless, P11’s disinterest caused a quick answering of the questionnaires, which might be the reason for the low objective understanding scores. This persona highlights a user who is perforce engaged but unconvinced of the system’s utility.
• “Satisfied mid-adopter”: P05 maintained a steady and moderate level of interaction with the system, using it consistently throughout the deployment. The user rated usability positively and expressed satisfaction with the robot, with statements such as “because the risk of a person falling is high, the more technologies to help detect them, the better” or “It frees workload in rush hours”. However, P05 believed that he/she understood the system well, but performance on the objective understanding questions suggested otherwise. This indicates a possible overestimation of comprehension, which did not affect the general satisfaction or usage. This user represents a persona who can operate the system while being moderately satisfied by its usability and usefulness.
• “Non-adopter”: P09 rarely interacted with the system, only doing so when explicitly required to address an alarm or fix an issue: “One night I had to return it to its base”. Nevertheless, P09 did not configure any patrols, likely because responsibility for configuration and other robot management was assumed by a more proactive colleague during the same shift: “I have not turned it on or off … my colleagues do that. I’ve only seen it patrolling”. The user reported low usage and perceived the system as less relevant to their workflow. Despite this low engagement, P09 displayed a good balance between subjective and objective understanding, suggesting that he/she was capable of using the system if necessary. This user represents a pragmatic non-adopter: someone who is minimally involved but sufficiently informed to use the technology when required, often as a result of external prompts rather than intrinsic motivation.

## 7 Discussion

In this section, we discuss the results provided above, drawing on general patterns observed from qualitative responses across all participants. Our analysis reveals several key themes that inform our understanding of longitudinal human-robot interaction in healthcare settings.

### 7.1 Understanding dynamics

Our findings reveal important discrepancies between subjective and objective understanding measures across several user participants. This mismatch indicates that users’ perceived comprehension increases more rapidly than their actual understanding as they become familiar with the system. While it remains unclear whether both metrics eventually reach stability—potentially due to staff shift changes and deployment interruptions—this pattern suggests that prolonged exposure to robotic systems may lead to overconfidence in one’s understanding.

Interestingly, participants sometimes demonstrated awareness of large knowledge gaps while simultaneously reporting moderate levels of subjective understanding. For instance, P01 stated “I have not experienced it, I have no idea” yet selected a score of 4 on the 1-to-7 subjective understanding scale. This phenomenon has been examined in greater detail in previous work, which provides a comprehensive analysis of objective and subjective understanding dynamics through additional sub-questions in the understanding questionnaire (Gebelli et al., 2025).

### 7.2 Iterative refinement and system issues

The final evaluation deployment revealed that participatory design is an ongoing process that extends beyond formal co-design sessions. During the first week of the two-month autonomous pilot, numerous participants provided suggestions for improvements, demonstrating that system evaluation inherently generates additional feedback for refinement, especially taking into account that they interact with a more advanced prototype for a longer time. Resource constraints and project timelines typically determine when this iterative process concludes, limiting the implementation of new improvements.

Some participant requests represented previously unidentified needs that were straightforward to implement. Examples include reducing screen brightness during night shifts after P11 complained “The screen is to bright during the night,” and introducing brief non-verbal audio greetings when entering rooms, as suggested by multiple participants: P05 noted “it should say hello before entering,” P06 stated “It would be great that it would greet, something very basic,” and P07 observed “People expect it to talk, not necessarily a conversation.”. As such, we quickly modified the system to dim the screen light at night and to play a sound before entering the patients’ rooms.

Other improvements mentioned by participants had been previously considered but discarded during earlier co-design phases due to technological or resource limitations required for autonomous operation. These included medication or food delivery to patients, with P10 commenting “It would be great if it would also be able to deliver medication or juice drinks,” and video calling capabilities, which P05 mentioned consistently across three consecutive weeks.

Performance-related concerns that could not be immediately addressed during deployment included navigation failures (P01: “The main issue is when it gets blocked, which happens often … ”), inability to detect falls while navigating in corridor areas (P03: “It would be nice to be able to detect falls in the corridor”), missed detections (P11: “it should […] and detect better people”), and phone usability issues (P04: “We carry many things in the pocket, it is annoying to carry an additional phone sometimes”).

Despite these performance issues in perception and navigation, usability and usage scores did not decrease significantly, confirming that system performance was sufficient for the intended tasks. While false alarms and robot malfunctions requiring intervention occurred more frequently than desired for a production system, participants’ understanding that this was a prototype, combined with shared workload management across shift teams, prevented system usage abandonment. In this direction, it was crucial that any person could dismiss false alarms or solve navigation issues, and that all other staff could continue their routine undisturbed. Combined with the fact that it took little time to resolve those situations, think-aloud responses suggest that participants were not exceedingly concerned with the extra time spent during the low-performance situations.

In case the prototype would be further developed into a performant final solution, two main root causes and solutions were identified. Regarding the perception system, the principal issue was inaccurate or nonexistent depth information. The solution would be to use a more precise depth camera, or if feasible, a 3D lidar. With respect to the navigation problems, recovery strategies should be additionally developed to further attempt to find new valid paths.

### 7.3 Placebo effects

Our observations suggest that some positive outcomes occurred despite the limited actual system performance, indicating potential placebo effects operating at multiple levels. Given the relatively low number of successful robot interventions and frequent false alarms, these effects warrant careful consideration.

Some participants’ responses informed about the patient’s potential placebo sentiment from the system, with P10 reporting “More than one patient has said that they feel accompanied, only one patient does not want it to come to the room” and P01 noting “In general, the patients like it, they feel watched over.” Many patients expressed satisfaction with the system and missed the visits on particular days “It has not come yet to see me this morning”.

Similar effects were observed among nursing staff, with participants reporting an enhanced sense of security. P10 stated “it gives us and the patients peace of mind”, similar to P03’s opinion “It gives you peace of mind.” Staff members appeared to derive reassurance from the robot’s presence and its potential to detect dangerous situations, regardless of actual detection performance.

Family members also seemed to react positively to the robot’s presence, though the robot was mostly idle when families were around, because the patients were already watched over by the families, and navigation became even more challenging. P07 noted “We use it when there are few families. They like it, especially children, but the robot cannot move well, and it becomes a problem for us”. Interestingly, family members frequently inquired, often jokingly, whether the robot was designed for floor cleaning or food delivery—the two service robot applications with which they were most familiar.

While participatory design helps to surface user needs and expectations, eliminating the placebo effect requires extended interactions, for periods even longer than our final evaluation. It is through prolonged, situated interaction (“learning by doing”) that users gradually recalibrate their expectations and understand the system’s actual capabilities. Our approach of conducting *in-situ* iterative deployments is intended to support this process. Unlike a one-off deployment, *in-situ* co-design enables the early identification of mismatches between perceived and actual system capabilities. Where feasible, the prototype can then be adapted to address genuine needs that were initially expressed through the placebo effect, but also to provide relevant feedback to make users aware of the real capabilities. This is tightly related to modifying the objective and subjective understanding, as discussed earlier in Section 7.1.

### 7.4 Impact of observation

The Hawthorne effect (Landsberger, 1958) states that humans have behaviour changes due to awareness of being observed, which has also been documented in HRI studies (Reimann et al., 2023; Belpaeme, 2020). This phenomenon likely affected our study in several ways.

Weekly questionnaire administration may have encouraged increased system usage, as participants anticipated being asked about their interactions. Additionally, social desirability bias (Grimm, 2010) may have influenced responses, with participants potentially reporting more favourable usage and usability ratings than their genuine assessments. The personal relationships developed with the research team through the participatory design process could have impacted this effect, though the direction of influence remains unclear–while participants may have felt more comfortable expressing criticisms due to the established bond, they may also have been reluctant to provide harsh feedback about a system developed by familiar researchers.

Participants also demonstrated curiosity about the questionnaire content, particularly regarding understanding assessments that remained consistent across time points. Especially the objective understanding part, which was single-choice *ABCDE* questions, might have been felt as an “exam” where they had to improve over time. P03 explicitly stated “This weekend, while being alone [with the robot], I’ll spend time trying things out”. This suggests that measurement activities themselves may have generated artificial engagement beyond genuine system-driven usage. Moreover, the feedback loops and final evaluation are unbalanced towards the participants who were working during more shifts during the study.

Finally, institutional support for the project by the hospital management may have created implicit pressure to use the system. P08 reflected this sentiment: “I am using it because we have to, and so it can patrol. Though at first, it’s kind of funny.” Such organisational influence represents an additional confounding factor in interpreting usage patterns and user acceptance metrics.

### 7.5 Limitations

While this study provides valuable insights into participatory design for assistive robotics in healthcare settings, several limitations must be acknowledged.

#### 7.5.1 Platform selection

The robotic platform (TIAGo) was predetermined rather than emerging from the participatory design process itself. This constraint may have influenced the scope of possible functionalities and limited the extent to which users could shape the fundamental characteristics of the system. Ideally, platform selection would be part of the co-design process, allowing stakeholders to contribute to decisions about form factor, mobility capabilities, and interaction modalities as proposed in Liberman-Pincu et al. (2024).

#### 7.5.2 Evolving system during evaluation

The system continued to undergo minor modifications throughout the deployment period to address robustness issues and incorporate small feature requests from users. While this iterative refinement reflects real-world deployment practices, the system being evaluated was not entirely static during the first weeks, which may affect the longitudinal analysis.

#### 7.5.3 Technical reliability challenges

The autonomous operation of the system was occasionally compromised by navigation failures and perception errors, requiring human intervention. These robustness concerns have probably influenced the results in ways that cannot be fully determined; however, as discussed in Section 7.2, usage did not decline over the 2-month deployment, indicating that the performance issues were not excessively severe. Technical limitations also necessitated the use of mobile applications for indirect interactions that might otherwise have been conducted through direct human-robot interaction. While participants shared physical space with the robot extensively, they could not engage in natural spoken dialogue with it, potentially affecting user acceptance patterns.

#### 7.5.4 Data collection constraints

The in-the-wild nature of the study, while ecologically valid, created significant challenges for systematic data collection. Healthcare staff were often too busy to complete questionnaires or participate in extended interviews, potentially introducing sampling bias toward periods of lower workload. Maintaining consistent participant engagement proved challenging due to staff holidays, sick leave, shift changes, and temporary personnel assignments. The timing of our study during summer holidays aggravated these issues, resulting in frequent changes to the participant pool. These issues limit the generalisation of the results, especially regarding the transfer to other healthcare contexts. Nevertheless, further iterations on the co-design process can help to adapt to other contexts, such as different patient types who require the monitoring of different characteristics. Additionally, the regular questionnaire administration and researcher presence may have influenced participant behaviour through the Hawthorne effects and social desirability biases, as already discussed in the previous subsection.

#### 7.5.5 Contextual disruptions

The introduction of palliative care patients and associated staff from another unit created significant contextual changes that influenced system usage patterns. These newcomers were unfamiliar with the robot and were managing more complex patient care scenarios, leading to reduced system engagement. While this represents a realistic healthcare environment challenge, it introduces confounding factors that complicate the interpretation of usage trends and user acceptance patterns.

Despite these limitations, the study demonstrates the feasibility and value of extended participatory design processes in healthcare robotics, providing a foundation for future research that might address these methodological challenges. The *in-situ* design and deployment of an autonomous system in a real-world context enhances the value and validity of the results, as they reflect participants’ authentic interactions with a system they could freely choose to use, and that was developed through a participatory design process tailored to their specific needs. Such extended autonomous deployments remain uncommon in the HRI community.

## 8 Conclusion and future work

This work presents a participatory design approach for developing assistive robots in healthcare settings, centred on iterative *in-situ* co-design to ensure context-based requirements gathering and system refinement. Our methodology addresses key limitations in traditional co-design processes by conducting design activities within the actual deployment environment, using low-fidelity prototypes to enable informed stakeholder participation throughout the whole process.

The implementation of this approach at the healthcare centre over 10 months demonstrated the feasibility and value of extended participatory design in real-world healthcare environments in a relatively short time. Through three main phases–observation and inspiration, *in-situ* co-design through prototyping, and evaluation–we successfully developed an autonomous patrolling robot that operated continuously across the final two-month autonomous deployment.

Our evaluation revealed several key findings. First, the system achieved sufficient robustness for autonomous operation in a complex healthcare environment, despite technical challenges including navigation failures and perception errors. Second, user acceptance patterns varied significantly across individuals, with five distinct user personas emerging: enthusiastic high-adopters, disillusioned high-adopters, unconvinced mid-adopters, satisfied mid-adopters, and non-adopters. Third, we observed important discrepancies between subjective and objective understanding measures, suggesting that prolonged exposure to robotic systems may lead to overconfidence in user comprehension.

Several areas warrant future investigation. The proposed participatory design methodology could be extended to include platform selection as part of the co-design process, allowing stakeholders to contribute to fundamental system characteristics beyond functionality. Technical improvements should focus on enhancing autonomous navigation reliability and perception accuracy. The integration of more sophisticated human-robot interaction capabilities, including natural language dialogue, could significantly improve user experience and system adoption. In terms of long-term operational viability, the current system remains at the prototype stage, and further work is required to enhance its technical robustness to support reliable use. Nonetheless, the underlying design process is intended to be sustainable, as the system can be adapted to similar tasks or changing contexts with few extra co-design iterations, which should continue to take into account privacy and ethical considerations as the solution evolves from a prototype to a permanent or commercial solution.

The findings of this work contribute to the growing body of knowledge on participatory design and longitudinal human-robot interaction studies, and provide practical guidance for researchers and practitioners developing assistive robotics systems for healthcare environments. By demonstrating that participatory design can successfully bridge the gap between technological capabilities and real-world user needs, this research advances our understanding of how to develop more effective and accepted robotic assistants for healthcare applications.

## Data Availability

The raw data supporting the conclusions of this article will be made available by the authors, without undue reservation.

## Footnotes

1
https://www.diligentrobots.com, accessed June 2025

2
https://static1.squarespace.com/static/606f1bb0f7e05e3329035ff8/t/646ea7519de79e7603715e12/1746482229015/AONL_Shannon_CaseStudy_v3.pdf, accessed June 2025

3
https://static1.squarespace.com/static/606f1bb0f7e05e3329035ff8/t/646ea7519de79e7603715e12/1746482229015/AONL_Shannon_CaseStudy_v3.pdf, accessed June 2025

4
https://www.fp-robotics.com/en/lio/, accessed June 2025

5 Note that within this work, we are solely considering co-design in robotics

6
https://ai.google.dev/edge/mediapipe/solutions/vision/pose_landmarker, accessed June 2025

7 The ethical committee from the hospital where the study was performed allowed the study’s execution. The requirement of ethical approval was waived by Badalona Serveis Assistencials (BSA) for the studies involving humans because it was considered an innovation project rather than a research study (March 2024).

8 The palliative unit was temporarily closed due to reduced staffing over the summer holiday period

9 In particular, the number of measurements was, chronologically, 16, 15, 14, 14, 17, 10, 15, 7 for each of the weeks
